# Supplementary material for: Gated Recurrent Units Viewed Through the Lens of Continuous Time Dynamical Systems
Source: Front Comput Neurosci. 2021 Jul 22;15:678158. doi: 10.3389/fncom.2021.678158 (PMC8339926; doi:10.3389/fncom.2021.678158)
Supplement: Supplementary file 1 [file Data_Sheet_1.PDF]

Supplementary Material

1 CONTINUOUS TIME SYSTEM DERIVATION

We begin with the fully gated GRU as a discrete time system, where the input vector x_t has been set equal to zero, as depicted in (S1) - (S3), where $\odot$ is the Hadamard product, and σ is the *sigmoid function*.

$$z_t = \sigma(U_z h_{t-1} + b_z) \tag{S1}$$

$$r_t = \sigma(U_r h_{t-1} + b_r) \tag{S2}$$

$$h_t = z_t \odot h_{t-1} + (1 - z_t) \odot \tanh\left(U_h(r_t \odot h_{t-1}) + b_h\right) \tag{S3}$$

We recognize that (S3) is a forward Euler discretization of a continuous time dynamical system. This allows us to consider the underlying continuous time dynamics on the basis of the discretization. The following steps are a walk through of the derivation:

Since z_t is a bounded function on $\mathbb{R}\ \forall t$, there exists a function $\tilde{z}_t$, such that $z_t + \tilde{z}_t = 1$ at each time step (due to the symmetry of z_t, $\tilde{z}_t$ is the result of vertically flipping z_t about 0.5, the midpoint of its range). As such, we can rewrite (S3) with $\tilde{z}_t$ as depicted in (S4).

$$h_t = (1 - \tilde{z}_t) \odot h_{t-1} + \tilde{z}_t \odot \tanh\left(U_h(r_t \odot h_{t-1}) + b_h\right) \tag{S4}$$

where,

$$\tilde{z}_t = \sigma(\tilde{U_z} h_{t-1} + \tilde{b_z}) \tag{S5}$$

$$h_t = h_{t-1} - \tilde{z}_t \odot h_{t-1} + \tilde{z}_t \odot \tanh\left(U_h(r_t \odot h_{t-1}) + b_h\right) \tag{S6}$$

$$h_t - h_{t-1} = -\tilde{z}_t \odot \left(h_{t-1} - \tanh\left(U_h(r_t \odot h_{t-1}) + b_h\right)\right) \tag{S7}$$

Let $h(t) \equiv h_{t-1}$. As a result, we can say $\tilde{z}_t \equiv \tilde{z}(t)$ and $r_t \equiv r(t)$, as depicted in (S8).

$$h(t+1) - h(t) = -\tilde{z}(t) \odot \left(h(t) - \tanh\left(U_h(r(t) \odot h(t)) + b_h\right)\right) \tag{S8}$$

where,

$$\tilde{z}(t) = \sigma(\tilde{U_z} h(t) + \tilde{b_z}) \tag{S9}$$

$$r(t) = \sigma(U_r h(t) + b_r) \tag{S10}$$

Let Δt define an arbitrary time interval. Then (S8) becomes,

$$h(t+\Delta t) - h(t) = -\tilde{z}(t) \odot (h(t) - \tanh U_h(r(t) \odot h(t)) + b_h)\Delta t \tag{S11}$$

Dividing both sides of the equation by Δt yields (S12).

$$\frac{h(t+\Delta t) - h(t)}{\Delta t} = -\tilde{z}(t) \odot (h(t) - \tanh U_h(r(t) \odot h(t)) + b_h) \tag{S12}$$

If we take the limit as $\Delta t \to 0$, we get the analogous continuous time system to (S1) - (S3),

$$\dot{h} = -\tilde{z}(t) \odot \left(h(t) - \tanh\left(U_h(r(t) \odot h(t)) + b_h\right)\right) \tag{S13}$$

where $\dot{h} \equiv \frac{dh(t)}{dt}$

Finally, we can rewrite (S13) as follows:

$$\dot{h} = (z(t) - 1) \odot (h(t) - \tanh\left(U_h(r(t) \odot h(t)) + b_h\right)) \tag{S14}$$

where

$$z(t) = \sigma(U_z h(t) + b_z) \tag{S15}$$

2 SINGLE GRU FIXED POINT PROOFS

The fixed points of our continuous time system (S13) exist where the derivative $\dot{h} = 0$. In the single GRU case, the Hadamard product reduces to standard scalar multiplication, yielding,

$$0 = (z(t) - 1)[h^* - \tanh\left(U_h r(t) h^* + b_h\right)] \tag{S16}$$

where $z(t)$ and $r(t)$ are defined by (S15) and (S10) respectively, and $h^* \in \mathbb{R}$ represents a solution of (S16).

We can divide out $z(t) - 1$, indicating that the update gate does not play a part in the stability of the system. For simplicity, lets expand $r(t)$ in (S16) by its definition (S10).

$$0 = \tanh\left(U_h \sigma(U_r h^* + b_r) h^* + b_h\right) - h^* \tag{S17}$$

where $U_h, b_h, U_r, b_r \in \mathbb{R}$.

LEMMA 1. *There exists a set of parameters U_h, b_h, U_r, b_r such that there exists one, two, or three solutions to* (S17).

PROOF. We will prove this lemma by showing the existence of each case. Let $U_r = 80$, $b_r = 40$, and $U_h = -60$. We then allow b_h to vary. The existence of each of the three cases are shown in Fig. 1 of the main text.

If $b_h = 1$, there exists a single solution to (S17). If b_h decreases continuously, a second root appears and splits in two. Analogously, the system (S13) goes through a *saddle-node bifurcation*, where a half-stable fixed point appears, and splits into a stable/unstable fixed point pair.

THEOREM 1. *For any choice of parameters U_r, b_r, U_h, b_h, there can only exist one, two, or three solutions to* (S17)*, and all solutions exist on the interval (-1,1).*

PROOF. We begin with the argument of the hyperbolic tangent function in (S17),

$$U_h \sigma(U_r h + b_r) h + b_h \tag{S18}$$

Taking the derivative of (S18) yields,

$$\frac{U_h(1 + e^{-U_r h - b_r}) + U_h U_r h e^{-U_r h - b_r}}{(1 + e^{-U_r h - b_r})^2} \tag{S19}$$

Setting (S19) to zero and simplifying will allow us to find the nontrivial critical points of (S18), as shown in (S20). Note that if $U_h = 0$, (S18) is equal to b_h $\forall h$, yielding no critical points.

$$1 + e^{-U_r h - b_r}(1 + U_r h) = 0 \tag{S20}$$

Let $x \equiv e^{-b_r}$ and $\hat{h} \equiv U_r h$, and solve (S20) for $\hat{h}$.

$$\hat{h} = -W(\frac{1}{xe}) - 1 \tag{S21}$$

where W is principal branch of the *Lambert W function*. Therefore, (S18) has exactly one local maximum or minimum, so long as $U_h \neq 0$.

Now consider,

$$\tanh\left(U_h \sigma(U_r h + b_r)h + b_h\right) \tag{S22}$$

The hyperbolic tangent function preserves intervals of monotonic behavior in its argument. Therefore, (S22) has at most one local maximum or minimum.

We take into account the fact that the hyperbolic tangent function bounds its argument on the interval (-1,1). If there exists a subset $S = [a, 1]$, for some $a \in [-1, 1)$ such that (S22) is increasing, then there exists a $k \in S$ such that when $h = k$ (S23) and (S24) hold.

$$\frac{d}{dh}(\tanh\left(U_h \sigma(U_r h + b_r)h + b_h\right) - h) = 0 \tag{S23}$$

$$\frac{d}{dh}(\tanh\left(U_h \sigma(U_r h + b_r)h + b_h\right) - h) < 0, \forall h > k \tag{S24}$$

This result in conjunction with the previous lemma completes the proof.

3 ALL OBSERVED NONTRIVIAL 2-D GRU FIXED POINT STRUCTURES

Fig. S1 lists all the parameters used for the phase portraits in Section 4 in the main text of this manuscript. Fig. S2 depicts all observed topologies of multiple-fixed point structures using 2D GRUs. Figs. S3 –S5 displays an example of a phase portrait from a 2D GRU system for each case listed in Fig. S2. Note that all fixed points are denoted by a red dot, regardless of classification. Fig. S6 lists the parameters used for each of the observed cases. Note that all the update gate parameters are set to zero.

Each case in this paper was discovered by hand by considering the geometric constraints on the structure of nullclines for both the decoupled and coupled system (i.e reset gate inactive and active respectively). An exhaustive analysis on the one dimensional GRU allowed for a natural extension into the two dimensional decoupled GRU. Upon establishing a set of base cases, the reset gate can be used as a means of bending and manipulating structure of the decoupled nullclines in order to obtain new intersection patterns in the coupled system.

**Fig**	**Uh11**	**Uh12**	**Uh21**	**Uh22**	**Ur11**	**Ur12**	**Ur21**	**Ur22**	**bh1**	**bh2**	**br1**	**br2**
**2**	3	0	0	3	0	0	0	0	0	0	0	0
**3a**	2	0	0	2	5	8	8	5	0	0	5	5
**3b**	2	0	0	2	-1	0	0	-1	0	0	0	0
**3c**	2	0	0	2	1	-2	3	1	-0.06	0	0.2	-0.85
**9a**	2	0	0	2	5	9	5	9	0	0	0	0
**9b**	2	0	0	2	5	9	9	5	0	0	0	0
**10**	0.1	-0.1	-1	0	0	0	0	0	0	0	0	0

Table 1. All previously unspecified parameters used for all phase portraits in Section 4

Figure S1: All unspecified parameters used for phase portraits in Section 4

Case	Fixed Points	Sinks	Sources	Saddle Points	Saddle Point and Stable Node Collisions	Saddle Point and Unstable Node Collisions	Codim. 2 Bifurcation Point
i	2	1	-	-	1	-	-
ii	3	2	-	1	-	-	-
iii	3	1	-	-	2	-	-
iv	4	1	-	-	2	-	1
v	4	2	-	1	-	-	1
vi	4	2	-	1	-	1	-
vii	4	2	-	1	1	-	-
viii	4	1	-	-	3	-	-
ix	5	2	1	2	-	-	-
x	5	3	-	2	-	-	-
xi	5	3	-	1	-	1	-
xii	5	2	-	1	-	2	-
xiii	5	2	-	1	1	1	-
xiv	5	-	1	-	4	-	-
xv	6	2	-	1	2	1	-
xvi	6	3	-	2	-	1	-
xvii	6	2	1	2	1	-	-
xviii	6	3	-	2	-	-	1
xix	6	2	1	2	-	1	-
xx	6	3	-	2	1	-	-
xxi	6	1	1	1	3	-	-
xxii	7	3	1	3	-	-	-
xxiii	7	2	2	3	-	-	-
xxiv	7	4	3	-	-	-	-
xxv	7	2	1	2	2	-	-
xxvi	7	3	-	2	2	-	-
xxvii	7	3	-	2	1	1	-
xxviii	8	4	-	3	-	1	-
xxix	8	3	1	3	1	-	-
xxx	8	3	-	2	2	1	-
xxxi	9	4	1	4	-	-	-
xxxii	9	3	1	3	2	-	-
xxxiii	9	5	-	4	-	-	-
xxxiv	10	4	1	4	1	-	-
xxxv	10	5	-	4	-	1	-
xxxvi	11	5	1	5	-	-	-

Figure S2: Multiple Fixed Point Stability Structures Obtainable with 2D GRUs

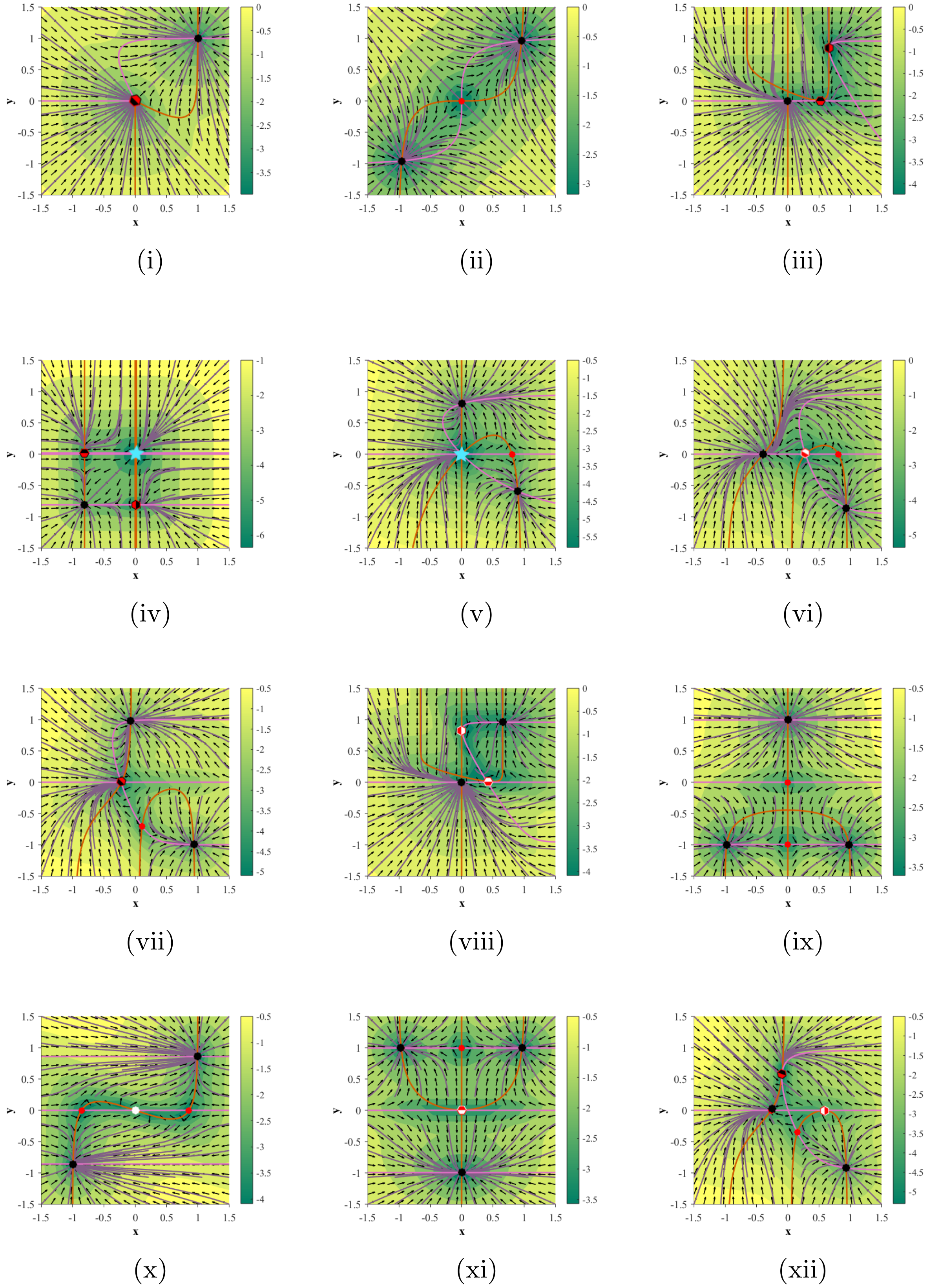

Figure S3: Thirty six multiple fixed-point topologies obtainable with 2D GRUs, depicted in phase space. Orange and pink lines represent the x and y nullclines respectively. Each subfigure contains 64 purple lines, indicating trajectories in forward time, whose initial conditions were chosen to be evenly spaced on the vertices of a square grid on $[-1.5, 1.5]^2$. Direction of the flow is determined by the black arrows, and the underlying color map represents the magnitude of the velocity of the flow in log scale.

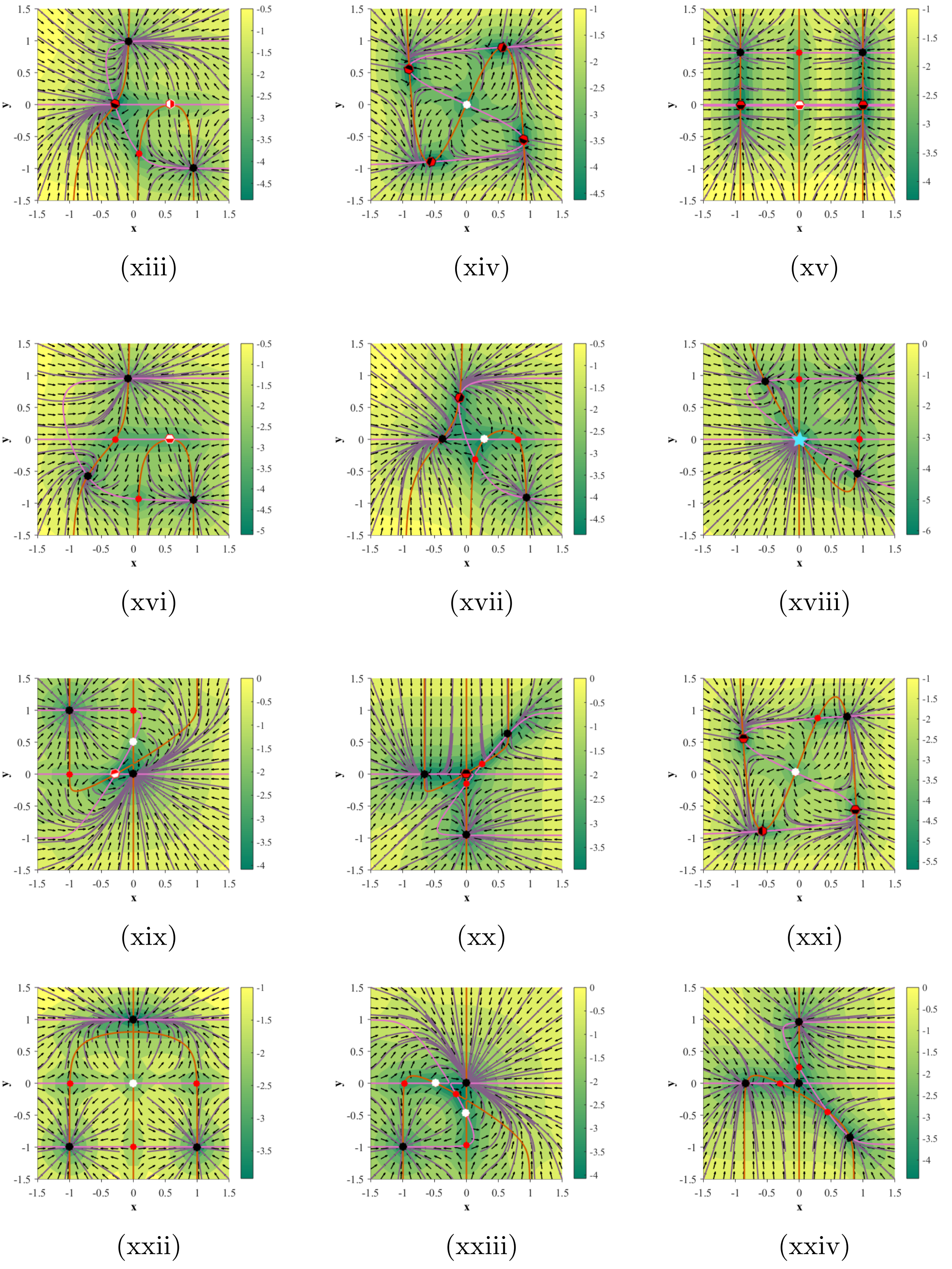

Figure S4: Thirty six multiple fixed-point topologies obtainable with 2D GRUs, depicted in phase space. Orange and pink lines represent the x and y nullclines respectively. Each subfigure contains 64 purple lines, indicating trajectories in forward time, whose initial conditions were chosen to be evenly spaced on the vertices of a square grid on $[-1.5, 1.5]^2$. Direction of the flow is determined by the black arrows, and the underlaying color map represents the magnitude of the velocity of the flow in log scale.

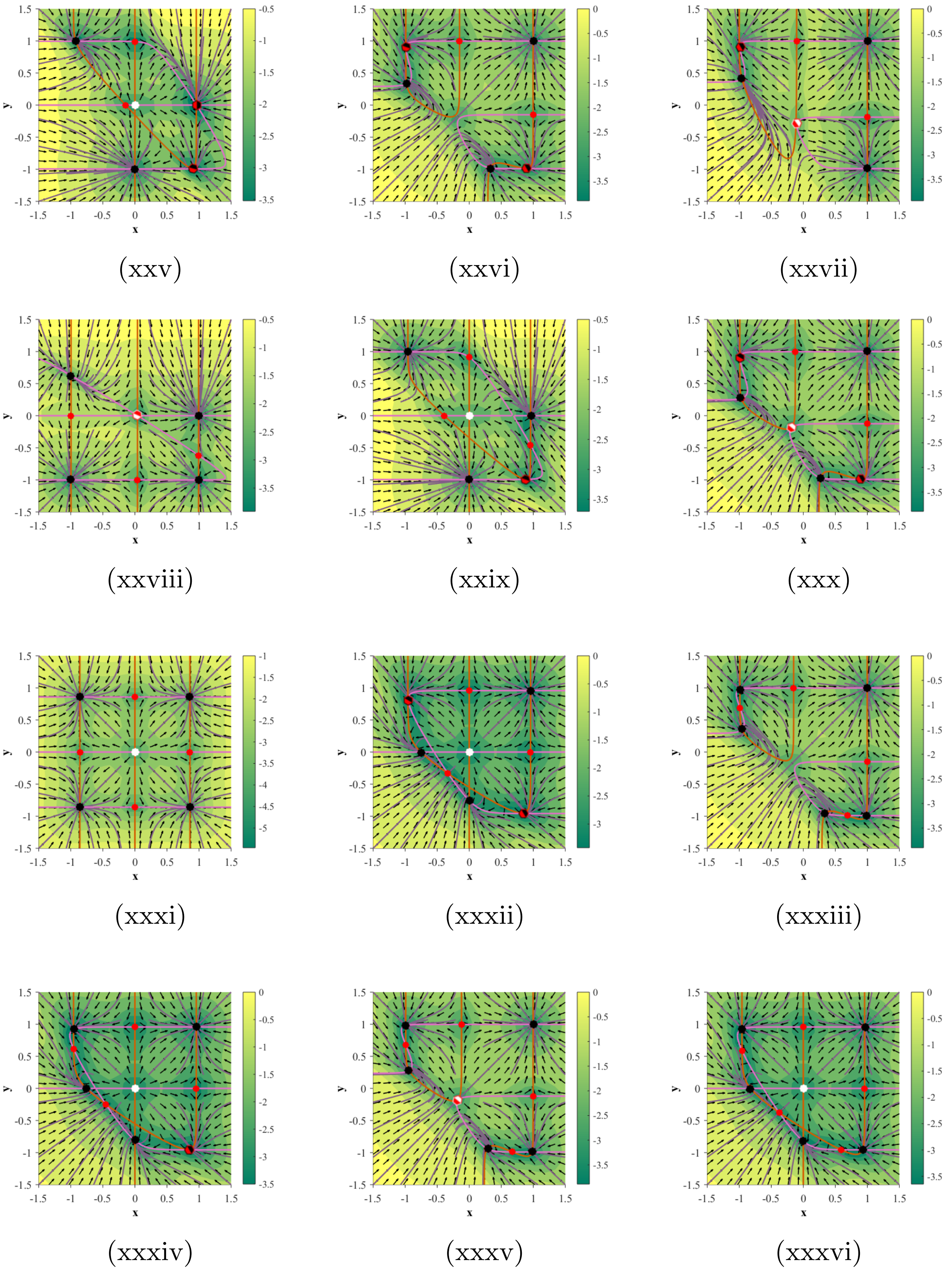

Figure S5: Thirty six multiple fixed-point topologies obtainable with 2D GRUs, depicted in phase space. Orange and pink lines represent the x and y nullclines respectively. Each subfigure contains 64 purple lines, indicating trajectories in forward time, whose initial conditions were chosen to be evenly spaced on the vertices of a square grid on $[-1.5, 1.5]^2$. Direction of the flow is determined by the black arrows, and the underlaying color map represents the magnitude of the velocity of the flow in log scale.

Case	Uh11	Uh12	Uh21	Uh22	Ur11	Ur12	Ur21	Ur22	bh1	bh2	br1	br2
i	2	2	2	2	100	100	100	100	0	0	0	0
ii	2	2	2	2	0	0	0	0	0	0	0	0
iii	1.2	0	0	2	5	12	8	5	0	0	-0.22	-0.5
iv	2	0	0	2	-1	0	0	-1	0	0	0	0
v	2	0	0	2	1	-1	1	1	0	0	0	0
vi	2	0	0	2	1	-2	3	1	-0.06	0	0.2	-0.85
vii	2	0	0	4	1	-2	3	1	-0.06	0	-0.3	-0.42
viii	1.2	0	0	2	5	20.5	8	5	0	0	-0.275	-3.3
ix	3	3	0	3	0	0	0	0	0	0	0	0
x	6	0	0	6	0	-2	0	0	0	0	-1.695	0
xi	6	0	0	6	0	1	0	0	0	0	-2.5	0
xii	2	0	0	2	1	-2	3	1	-0.055	0	-0.1	-0.02
xiii	2	0	0	4	1	-2	3	1	-0.06	0	-0.085	-0.22
xiv	2.9674	-0.4409	0.4409	2.9674	0	0	0	0	0	0	0	0
xv	6	0	0	2	-1	0	0	0	0	0	0	0
xvi	2	0	0	2	1	-2	3	1	-0.06	0	-0.08	3
xvii	2	0	0	2	1	-2	3	1.1	-0.06	0	0.2	0
xviii	2	0	0	2	3	2	2	3	0	0	0	0
xix	10	0	0	6	-3	5	-5	3	0	0	-3	-3
xx	1.2	0	0	2	-17	35	10	-8	0	0	1.2	-1.2
xxi	2.9763	-0.376	0.376	2.9763	0	0	0	0	0.0315	0	-0.015	-0.015
xxii	6	0	0	6	0	-2	0	0	0	0	0	0
xxiii0	3	0	0	3	-3	-5	-5	-3	0	0	-2	-2
xxiv	1.5	0	0	2	-4	-7	8	5	0	0	-0.4	-1.2
xxv	2	0	0	3	12.4	11.6	-8	-5	0	0	1.8	7
xxvi	3	0	0	3	5.175	9	9	5.175	0.3	0.3	3.95	3.95
xxvii	7	0	0	3	6	3	9	6	0.62	0.373	4	3.4
xxviii	6	0	0	10	0	0	-5	-8	-0.08	0	0	-2
xxix	2	0	0	3	-10	-11.6	8	5	0	0	4	4.8
xxx	3	0	0	3	5.26	9	9	5.26	0.25	0.25	3.95	3.95
xxxi	3	0	0	3	0	0	0	0	0	0	0	0
xxxii	2	0	0	2	5	8	8	5	0	0	4.4	4.4
xxxiii	3	0	0	3	6	9	9	6	0.3	0.3	3.75	3.75
xxxiv	1	0	0	1	5	8	8	5	0	0	4.4	4.9
xxxv	3	0	0	3	6	9	9	6	0.24	0.24	3.95	3.95
xxxvi	2	0	0	2	5	8	8	5	0	0	5	5

Figure S6: Parameters of each multiple fixed-point stability structure example
